# Cooperative Interaction between Acid and Copper Resistance in *Escherichia coli*

**DOI:** 10.4014/jmb.2201.01034

**Published:** 2022-03-06

**Authors:** Yeeun Kim, Seohyeon Lee, Kyungah Park, Hyunjin Yoon

**Affiliations:** 1 Department of Molecular Science and Technology, Ajou University, Suwon 16499, Republic of Korea; 2 Department of Applied Chemistry and Biological Engineering, Ajou University, Suwon 16499, Republic of Korea

**Keywords:** *Escherichia coli*, foodborne pathogen, acid resistance, copper resistance

## Abstract

The persistence of pathogenic *Escherichia coli* under acidic conditions poses a serious risk to food safety, especially in acidic foods such as kimchi. To identify the bacterial factors required for acid resistance, transcriptomic analysis was conducted on an acid-resistant enterotoxigenic *E. coli* strain and the genes with significant changes in their expression under acidic pH were selected as putative resistance factors against acid stress. These genes included those associated with a glutamatedependent acid resistance (GDAR) system and copper resistance. *E. coli* strains lacking GadA, GadB, or YbaST, the components of the GDAR system, exhibited significantly attenuated growth and survival under acidic stress conditions. Accordantly, the inhibition of the GDAR system by 3-mercaptopropionic acid and aminooxyacetic acid abolished bacterial adaptation and survival under acidic conditions, indicating the indispensable role of a GDAR system in acid resistance. Intriguingly, the lack of *cueR* encoding a transcriptional regulator for copper resistance genes markedly impaired bacterial resistance to acid stress as well as copper. Conversely, the absence of YbaST severely compromised bacterial resistance against copper, suggesting an interplay between acid and copper resistance. These results suggest that a GDAR system can be a promising target for developing control measures to prevent *E. coli* resistance to acid and copper treatments.

## Introduction

A diet that includes fresh or minimally processed vegetables is generally considered to provide lots of health benefits. However, in view of bacterial circulation in ecosystems, where bacterial pathogens are excreted to soil and water through animal feces and subsequently infect animal hosts by ingestion of contaminated food resources, farm produce contaminated with these pathogens serves as a vehicle conveying them to humans and animals. Accumulating evidence has revealed that numerous foodborne outbreaks were attributable to vegetables and fruits contaminated with *Salmonella*, *E. coli*, *Listeria*, and so on [1, 2]. Meanwhile, fermented vegetables enriched with lactic acid bacteria (LAB) are considered microbiologically safe due to the acidity and bacteriocins produced by LAB [3]. However, an increasing number of foodborne outbreaks are caused by fermented foods such as kimchi. Recently in Korea, a succession of outbreaks occurred as a result of kimchi contaminated with pathogenic *E. coli* [4, 5]. Although *E. coli* is commonly detected in the normal gut microbiota, some *E. coli* pathotypes including enteroaggregative *E. coli* (EAEC), enterohemorrhagic *E. coli* (EHEC), enteroinvasive *E. coli* (EIEC), enteropathogenic *E. coli* (EPEC), enterotoxigenic E.coli (ETEC), and diffusely adherent *E. coli* (DAEC) can cause fatal diarrheal diseases in humans [6, 7].

Enteric bacteria such as *E. coli* and *Salmonella* spp. are neutralophiles with a preference for neutral pH levels. However, during their life cycle they often encounter harsh and mild acidic environments such as those found in the human gastrointestinal tract (where pH ranges from 1.5-3.5 in the stomach to 4.0-6.0 in the small intestine), hygienic processing in the food industry, the traditional fermentation of foods, and the acid soils, and have developed multiple protective mechanisms against acidic stress in their evolution [8]. For example, *E. coli* maintains cellular proton concentrations below a lethal level by pumping out excessive protons via the F_1_F_0_ ATP synthase, or by consuming protons via amino acid-mediated decarboxylation reactions [8, 9]. *E. coli* also employs amino acid deiminases to produce ammonia, which in turn sequesters protons to generate ammonium ion [10]. Moreover, the phospholipid moiety of the cellular membrane is modified to reduce the permeability to extracellular protons [11] and chaperon proteins participate in protecting and repairing denatured proteins [12]. Especially in response to extreme acid stress, *E. coli* has evolved five different molecular mechanisms, called acid resistance (AR) systems. Among these, the AR1 system is activated by alternative sigma factor σ^S^, also known as σ^38^ or RpoS. σ^S^ increases its cellular level under varied stressful conditions including nutrient starvation and acidic pH and regulates the expression of multiple genes associated with stress resistance, including AR2 system genes [13, 14]. The AR2-AR5 systems comprise distinct pairs of a decarboxylase and an antiporter, where the decarboxylase enzyme consumes a proton and conversely removes the α-carboxyl group from glutamate/glutamine (AR2), arginine (AR3), lysine (AR4), and ornithine (AR5), respectively, leaving as CO_2_ and the antiporter exchanges the amino acid and the reaction by-product across the membrane [8, 15].

Among the five AR systems, the glutamate/glutamine-dependent AR2 system (or GDAR system) is the most proficient at neutralizing excessive protons [16] and its transcriptional regulatory network has been intensively characterized. GadE is a central regulator of the AR2 network and the expression of *gadE* is coordinated by multiple regulators binding to the large intergenic region upstream of *gadE*, including GadE itself, GadX, GadW, EvgA, YdeO, and MnmE [17]. GadE directly activates the transcription of *gadAB* and *gadC* genes, which encode the glutamate decarboxylase (GAD) isozymes (GadA and GadB) and their cognate antiporter GadC, respectively. GAD using pyridoxal phosphate (PLP) as a cofactor catalyzes the conversion of glutamic acid and proton to γ-aminobutyric acid (GABA) and CO_2_ [18]. GadC, which exchanges extracellular glutamate with intracellular by-product GABA, also exchanges extracellular glutamine with intracellular glutamate and cooperates with glutaminase YbaS to generate ammonia, while supplementing the intracellular glutamate pool [10]. In the *E. coli* chromosome, *ybaS* gene composes an operon with *ybaT* encoding an amino acid permease and this operon is localized between *copA* and *cueR*, which encode a copper (Cu^1+^) efflux pump, CopA, and a Cu^1+^-sensing transcriptional regulator CueR, respectively. The *cue/cop* regulon encoding the Cue system is required for exporting excessive Cu^1+^ from the bacterial cytoplasm and is known as a primary defense system against copper stress [19]. A recent study demonstrated that the expression of *ybaST* was induced by copper stress under acidic conditions and suggested a link between copper and acid resistance in *E. coli* [20].

In this study, a transcriptomics approach was employed to understand the comprehensive transcriptional response of *E. coli* to extreme acid stress and identify acid resistance factors, which can be further exploited for developing control measures against acid-resistant pathogenic *E. coli*.

## Materials and Methods

### Bacterial Strains

Enterotoxigenic *Escherichia coli* str. 4032 (hereafter, ETEC str. 4032) was isolated from kimchi in Korea and used in transcriptomic analysis. *E. coli* str. K-12 substr. MG1655 [21] (hereafter, *E. coli* MG1655) was used as a parent strain to construct mutant strains. All the bacterial strains used in this study are listed in Table 1. Mutant strains were all constructed using the λ Red recombination system [22, 23]. In brief, PCR products containing a kanamycin (*kan*) resistance cassette in the middle were generated using pKD13 as a template. For homologous recombination, the PCR primers were designed to amplify the *kan* cassette with 40-nucleotide flanking sequences homologous to target genes at both termini. Purified PCR products were introduced into *E. coli* MG1655 harboring Red helper plasmid pKD46. Recombinant cells were selected on *kan* plate and subsequently confirmed by diagnostic PCR. Afterwards, the *kan* cassette was removed by introducing pCP20, which produces the FLP recombinase [24]. All primers used for the construction of bacterial strains are listed in Table S1. Bacteriophage P1-mediated transduction was conducted to transfer genetic alleles marked with antibiotic resistance cassettes between bacterial strains as described [25]. Antibiotics were used as follows: ampicillin 50 μg/ml, kanamycin 50 μg/ml, chloramphenicol 35 μg/ml Sigma-Aldrich (Korea).

### Plasmid Construction

All the plasmids used in this study are listed in Table 1. Plasmids including pGadA, pGadB, pYbaS, pYbaST, and pCueR were constructed using pBAD18, pBAD30, or pBAD33 [26]. Genes of *gadA*, *gadB*, and *cueR* were amplified using PCR and cloned into pBAD30 [26], using XbaI/HindIII (*gadA* and *gadB*) and EcoRI/XbaI (*cueR*) restriction enzyme recognition sites. Genes of *ybaS* and *ybaST* were amplified by PCR, digested using XbaI/HindIII, and cloned into pBAD18 [26]. Three genetic loci of *cueR*, *ybaS*, and *ybaST* were also subcloned into pBAD33 [26]. Primers used for plasmid construction are listed in Table S1.

### Bacterial Growth Conditions and Acid Treatments

Bacterial cells were cultivated in Luria-Bertani (LB) broth Becton, Dickinson and Company (USA) or M9 minimal medium broth at 37°C, unless otherwise specified. M9 minimal medium broth was formulated as follows: 12.8 g/l Na_2_HPO_4_∙7H_2_O, 3 g/l KH_2_PO_4_, 0.5 g/l NaCl, 1 g/l NH_4_Cl, 0.24 g/l MgSO_4_, 1.8 g/l glucose. For transcriptomic analysis, ETEC str. 4032 was pre-cultured to the stationary growth phase in LB broth and diluted to fresh LB broth adjusted to pH 2.5 and pH 7.0, respectively. Bacterial cells were incubated for 30 min and used in RNA preparation. LB broth was acidified using acetic acid solution (Korea). To test the growth inhibitory effects of chemical compounds including 3-mercaptopropionic acid, aminooxyacetic acid, 4-deoxypyridoxine hydrochloride, isoniazid, and thiosemicarbazide, *E. coli* MG1655 cells were pre-cultured in pH 7.0 M9 minimal medium broth overnight and diluted into pH 7.0 or pH 5.5 M9 minimal medium broth at a ratio of 1:100 or 1:20. Acidity in M9 minimal medium broth was adjusted using HCl. Optical density at 600 nm (OD_600_) was measured to compare bacterial growth. All supplemented compounds were purchased from Sigma-Aldrich (Korea).

### RNA Extraction and RNA-Sequencing (RNS-Seq)

*E. coli* cells were treated with RNA Protect Bacterial Reagent (Qiagen, Germany) and total bacterial RNAs were isolated using an RNeasy Mini Kit (Qiagen) according to the manufacturer’s instructions. Genomic DNAs were removed using an Ambion Turbo DNA-free Kit (Ambion, USA) and an Agilent 2100 Bioanalyzer (Agilent Technoloies, USA) was employed to examine the quality and quantity of the total RNAs. The mRNA library was constructed using the Illumina TruSeq RNA Sample Preparation Kit v.2 (Illumina, USA) and RNA-Seq was performed by two runs of Illumina Hiseq to generate single-end reads around 100 bp in length. Using the CLRNAseq program (Chunlab, Korea), sequencing reads were mapped to the genome sequence of *E. coli* ETEC str. H10407 (Accession no. FN649414.1) (Table S2). RNA-Seq data were normalized using Reads Per Kilobase of transcript per Million mapped reads (RPKM), Relative Log Expression (RLE), and Trimmed Mean of M-value (TMM) [27-29] and RLE was applied for subsequent analyses, due to the lowest coefficient of variation (CV) value. Differentially expressed genes (DEGs) with a log_2_ [fold change] larger than 6 or smaller than -6 were sorted, where log2 [fold change] was defined as log2 [RLE_pH 2.5_/RLE_pH 7.0_]. The expression changes were displayed using heatmaps generated using Gitools v2.2.2 [30].

### Quantitative Reverse Transcription PCR (RT-qPCR)

*E. coli* strains were pre-cultured in pH 7.0 M9 minimal medium broth overnight and diluted into fresh M9 minimal medium broth at pH 7.0 and 5.5 as described above. After 3 h incubation, RNA degradation was quenched with RNA Protect Bacterial reagent and total RNAs were isolated using the RNeasy Mini Kit. After Ambion Turbo DNA-free treatments, cDNA was synthesized using RNA to cDNA EcoDry Premix (Random Hexamers) (Takara, Japan). RT-qPCR was conducted by using the StepOnePlus Real-Time PCR system (Applied Biosystems, Waltham, USA) with Power SYBR Green PCR Master Mix (Applied Biosystems). Ct values of target genes were normalized using those of *rpoD* encoding the housekeeping sigma factor (σ^70^) [31]. All primers used in RT-qPCR analysis are listed in Table S3.

### Bacterial Viability Assay

Bacterial survival under extremely acidic conditions was assayed as previously described with minor modifications [32]. *E. coli* strains were cultured in LBG (LB supplemented with 0.4% glucose) broth overnight and diluted into pH 5.5 LBG broth containing 0.012% L-glutamic acid. After 18 h incubation, bacterial cells were diluted at a 1:1000 ratio into fresh M9 minimal medium broth which was supplemented with 0.012% L-glutamic acid and acidified with HCl at pH 2.5. When required, the broth was supplemented with different concentrations of 3-mercaptopropionic acid and aminooxyacetic acid. For the expression of *gadA*, *gadB*, *ybaS*, *ybaST*, or *cueR* in trans, 0.2% L-arabinose was added in the culture. The number of live cells was determined at 2 h by diluting and spreading aliquots of the cultures onto LB agar plates. To evaluate bacterial resistance to copper, strains were pre-cultured in LB broth overnight and diluted into fresh M9 minimal medium broth supplemented with 40 μM or 50 μM CuSO_4_ (Sigma-Aldrich). L-arabinose at 0.2% was used to induce *ybaS*, *ybaST*, or *cueR* as described above. After 12 h incubation, viable cells were counted by plating bacterial cultures onto LB agar plates.

### Statistical Analysis

All experiments except RNA-Seq analysis were conducted in triplicate at least and the averaged values are expressed with standard deviations. *p*-values were calculated using one- or two-way analysis of variance with Tukey’s multiple comparison test. Statistical analysis was performed using GraphPad Prism 5 (GraphPad Software Inc., USA).

## Results

### Extreme Acid Stress Induced the Expression of AR2 System-Associated Genes

**Fig. 1:**
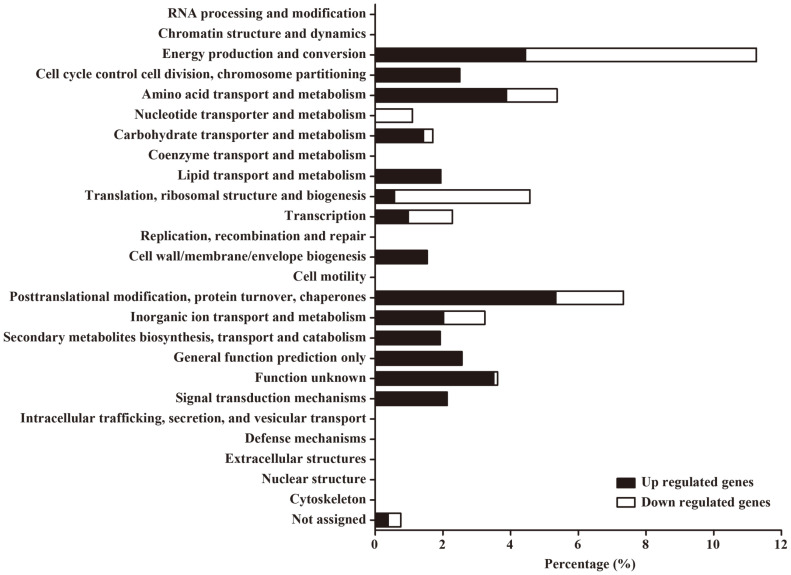
Functional categorization of DEGs. Genes with transcriptional changes (*p* < 0.05) in response to pH 2.5 treatments for 30 min were classified considering their predicted functions in cluster of orthologous group (COG) analysis. Genes upregulated (black bars) or downregulated (white bars) more than 6 folds were functionally grouped.

**Fig. 2:**
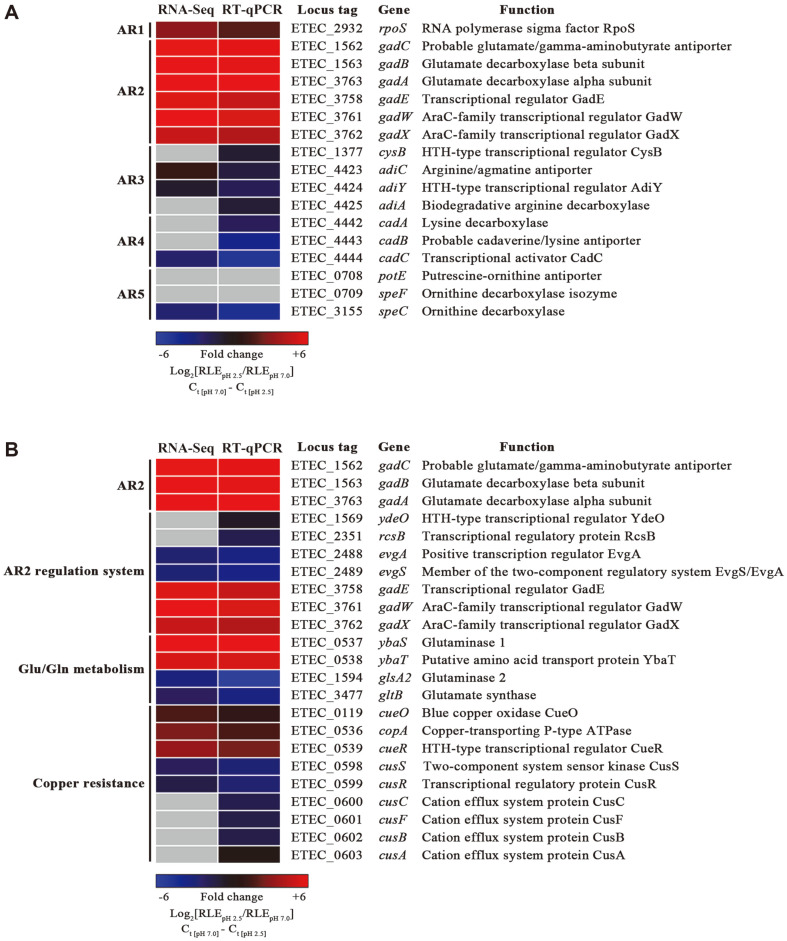
Expression of AR-associated genes in response to pH 2.5. (**A**) Genes associated with AR systems were sorted from the RNA-Seq data and their expression was compared between pH 2.5 and 7.0 and displayed using a heatmap, where the fold change of Log_2_[RLE_pH 2.5_/RLE_pH 7.0_] is shown in a colorimetric gradient. Genes not detected are shown in gray. The expression of AR-associated genes were reexamined using RT-qPCR and the expression ratio in ΔC_t_ is depicted in parallel: ΔC_t_ = C_t [pH 7.0]_ - C_t [pH 2.5]_, C_t_ values of each gene were normalized using those of a reference gene *rpoD*. (**B**) The transcription of AR2 system-relevant genes was compared between RNA-Seq and RT-qPCR results. The expression ratio between pH 2.5 and 7.0 was computed as described above and displayed in a colorimetric gradient.

To understand the physiological alterations of pathogenic *E. coli* under strong acid treatments, transcriptomic analysis was applied to an ETEC strain 4032 (ETEC str. 4032), which was isolated in Korea. ETEC str. 4032 cultivated in LB broth at a neutral pH level was exposed to LB broth titrated to pH 2.5 for 30 min and bacterial total RNAs were isolated and subjected to RNA-Seq analysis as described in Materials and Methods. DEGs showing transcriptional alteration more than six-fold upon acid treatments were sorted and listed in Table S4. DEGs included 102 upregulated genes and 46 downregulated genes and were categorized into 26 groups based on their predicted functions (Table S4, Fig. 1). ETEC str. 4032 showed significant transcriptional changes in genes associated with energy production and conversion (11.26%), posttranslational modification, protein turnover, chaperones (7.33%), and amino acid transport and metabolism (5.37%). The transcriptional alteration encompassing a wide range of cellular functions suggests that *E. coli* challenged by extreme acid stress orchestrated the transcriptional behavior of various genes required for bacterial adaptation and persistence under the hostile condition. As conative action to reconcile deleterious proton concentrations, *E. coli* harnesses five different AR systems from AR1 to AR5. ETEC str. 4032 exposed to pH 2.5 exhibited remarkable transcriptional increases in the genes associated with AR1 and AR2 systems, whereas genes relevant with AR3, AR4, and AR5 systems rarely changed their expression (Fig. 2A). The transcriptional changes of AR system genes were reexamined using RT-qPCR. In accordance with the RNA-Seq results, *rpoS* (AR1) and *gad* genes (AR2) were significantly increased in their transcription, but some genes associated with AR3, AR4, and AR5 systems showed rather decreased transcription under extreme acidic conditions (Fig. 2A). The differential transcription levels among AR system genes suggest that the AR1 and AR2 systems are primary defense devices for coping with extreme acid stress in ETEC str. 4032. The *E. coli* AR2 system (hereafter, GDAR system) is a complicated network comprising the products of multiple genes and the transcription levels of these cognate genes were examined in parallel (Fig. 2B). Genes encoding the substantial components (*gadABC*) of the GDAR system and their regulators (*gadEWX*) were all increased in their transcription in response to pH 2.5 and the auxiliary genes of *ybaST* also increased their transcription accordantly. However, other regulatory genes including *evgSA*, *ydeO*, and *rcsB* did not show transcriptional increases under the tested acidic condition. Intriguingly, the three genes of *cue/cop* regulon, which are transcribed as a divergent but contiguous operon with the *ybaST* locus in between, also exhibited transcriptional increases in response to extreme acid stress. These transcriptional parallels between *ybaST* and *cue/cop* regulon under acidic conditions implicate the possibility of the Cue system having a positive role in acid resistance.

### GDAR System and Cue System Are Resistance Factors Against Extreme Acid Stress

**Fig. 3:**
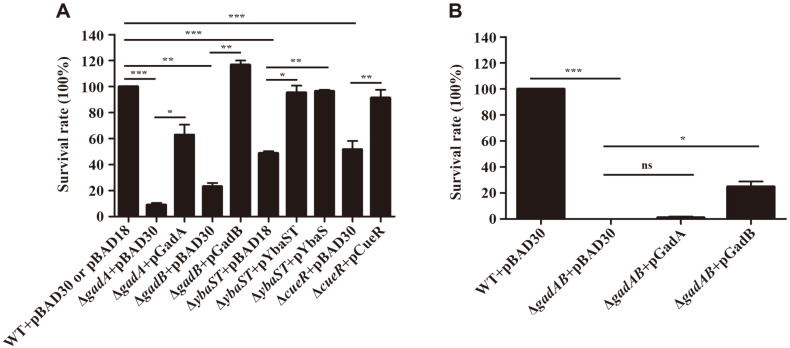
Viability of *E. coli* strains lacking GDAR system and Cue system under extremely acidic conditions. (**A**) Bacterial cells, which were pre-cultured overnight in pH 7.0 LBG, were adapted in pH 5.5 LBG for 18 h and transferred to pH 2.5 M9 minimal medium broth. After 2 h incubation, live cells were counted by plating onto LB agar. Bacterial survival rate was calculated by dividing the number (CFU/ml) at pH 2.5 by the number (CFU/ml) at pH 5.5 and the rate of wild-type *E. coli* was set to 100%. Tested bacterial strains include wild-type, Δ*gadA*, Δ*gadB*, Δ*ybaST*, and Δ*cueR* strains which were transformed with pBAD30 or pBAD18 or its derivative producing GadA, GadB, YbaS, YbaST, or CueR. (**B**) Acid resistance of *E. coli* wildtype and Δ*gadAB* strains harboring pBAD30, pGadA, or pGadB was tested as described above. Statistical significance is indicated by *, *p*-value < 0.05; **, *p*-value < 0.01; ***, *p*-value < 0.001.

The transcriptome analysis indicated that the genes associated with the GDAR and Cue systems promptly increased their transcription in response to extreme acid stress, suggesting their beneficial roles in acid resistance. The role of these genes in bacterial survival against extreme acid stress was investigated in *E. coli* using *E. coli* MG1655 as a model organism. As expected, the genes associated with the GDAR and Cue systems, including *gadABC*, *gadEWX*, *ybaST*, *cueR*, and *copA*/*cueO*, showed transcriptional increases in response to acidic stress in *E. coli* MG1655 as well as ETEC str. 4032 (Fig. S1). Interestingly, the expression levels of cusRS and cusCFBA constituting the cus system were rather decreased in both strains under acidic conditions. The cus system encodes an alternative tripartite copper efflux system, CusCBA, spanning the cell envelope [33]. To evaluate the importance of the GDAR and Cue systems in acid resistance, genes of *gadA*, *gadB*, and *ybaST* encoding the structural components of GDAR system and *cueR* encoding the regulator of the *cue/cop* regulon were deleted individually in *E. coli* MG1655 and bacterial survival under extreme acid treatments was compared. All bacterial strains including wild-type strain and mutant strains lacking *gadA*, *gadB*, *ybaST*, or *cueR* failed to grow when shifted from pH 5.5 to 2.5 in a minimal medium broth (Fig. S2). However, the numbers of live cells differed between wild-type and mutant strains (Fig. 3A). After 2 h incubation at pH 2.5, Δ*gadA*, Δ*gadB*, and Δ*ybaST* mutants showed 91, 77, and 51% reduction, respectively, in their survival, when compared with wild-type MG1655. The mutant lacking *cueR* also showed a significant decrease (48%) in its survival ability under acidic conditions. These results indicate that both the Cue system and the GDAR system are important for *E. coli* to survive under extremely acidic conditions. The attenuated survival in the absence of *gadA*, *gadB*, *ybaST*, and *cueR* was complemented totally or partially by introducing plasmids expressing the missing gene in each case (Fig. 3A).

*E. coli* possesses two homologous glutamate decarboxylase genes, *gadA* and *gadB*, showing 97% similarity in their sequences. *E. coli* devoid of both genes failed to resist strong acid stress, showing 0% survival, but the introduction of pGadB, which induces *gadB* under P_BAD_ promoter, restored bacterial viability in part, whereas pGadA failed to complement the survival defect of Δ*gadAB* mutant strain (Fig. 3B). This result suggests that GadB is more proficient in removing protons than GadA, as observed by others [34].

### 3-Mercaptopropionic Acid and Aminooxyacetic Acid Block Acid Resistance in *E. coli*

**Fig. 4:**
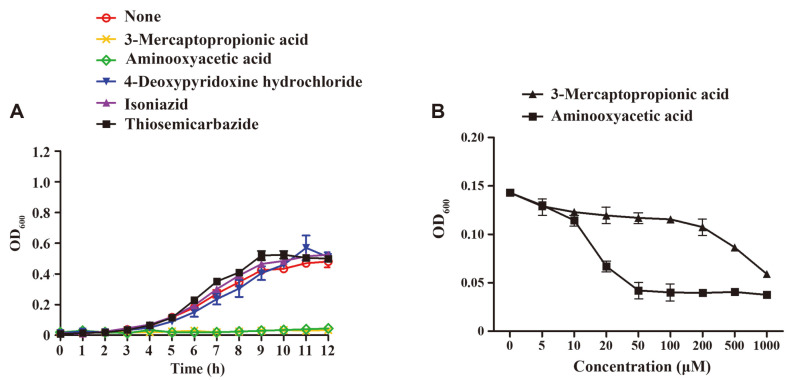
Screening compounds with inhibitory effects on bacterial growth under acidic conditions. (**A**) *E. coli* MG1655 was pre-cultivated in pH 7.0 M9 minimal medium broth and diluted to pH 5.5 M9 minimal medium broths supplemented with 5 different compounds including mercaptopropionic acid (0.5 mM), aminooxyacetic acid (0.02 mM), 4- deoxypyridoxine hydrochloride (1 mM), isoniazid (1 mM), and thiosemicarbazide (0.2 mM). The growth was measured for 12 h. (**B**) *E. coli* MG1655 pre-cultured in M9 minimal medium broth at pH 7.0 was diluted to pH 5.5 M9 minimal medium broth supplemented with 3-mercaptopropionic acid and aminooxyacetic acid at different concentrations from 0 μM to 1,000 μM. Bacterial growth was measured at 6 h and plotted.

**Fig. 5:**
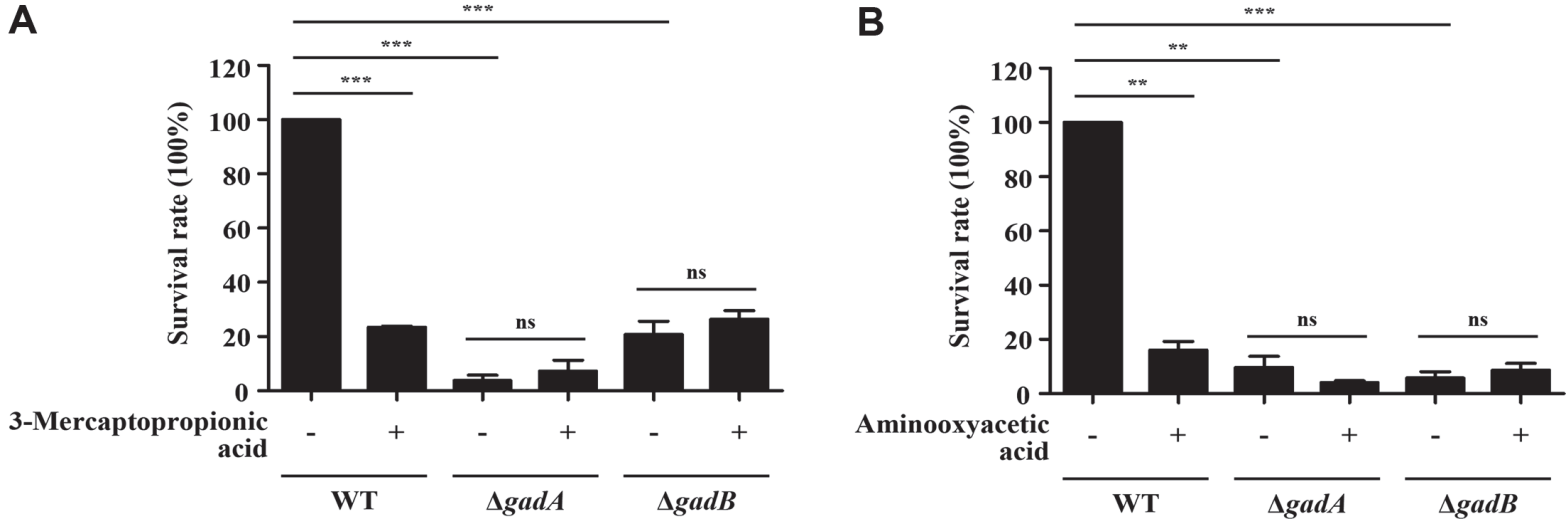
Effects of 3-mercaptopropionic acid and aminooxyacetic acid on the survival of *E. coli* under extremely acidic conditions. *E. coli* wild-type and mutant strains were cultivated in pH 5.5 LBG broth for 18 h and diluted to pH 2.5 M9 minimal medium broth containing 3-mercaptopropionic acid at 500 μM (**A**) or aminooxyacetic acid at 5 μM (**B**). After 2 h incubation, bacterial viability was determined by diluting and spreading aliquots of the culture samples onto LB agar plates. Bacterial survival rate was obtained by dividing the bacterial number (CFU/ml) from pH 2.5 samples by the number (CFU/ml) from pH 5.5 samples. The survival rate of wild-type *E. coli* was set to 100%.

Acid resistance was nearly abolished in *E. coli* lacking both *gadA* and *gadB*, indicating that a GDAR system is essential for bacterial survival against extreme acid stress. Chemical compounds that obstruct the activity of GadA and GadB can be an effective means to inhibit the development of bacterial resistance against extreme acid treatments. To test this possibility, five different compounds, presumed to play a role as either a substrate analogue or an antagonist in the GDAR system, were selected. These include 3-mercaptopropionic acid, which is an analogue of glutamate, and the other four, including aminooxyacetic acid, 4-deoxypyridoxine hydrochloride, isoniazid, and thiosemicarbazide, are antagonists of pyridoxal phosphate, a cofactor of GadA and GadB [35-42]. The concentrations of tested compounds were determined by measuring the maximal concentrations with no growth inhibitory effects at pH 7.0: 3-mercaptopropionic acid at 0.5 mM, aminooxyacetic acid at 0.02 mM, 4-deoxypyridoxine hydrochloride at 1 mM, isoniazid at 1 mM, and thiosemicarbazide at 0.2 mM. All tested compounds showed no inhibitory effects under pH 7.0 (Fig. S3). Each compound was added in M9 minimal medium broth adjusted to pH 5.5 and the growth of wild-type *E. coli* was compared (Fig. 4A). Bacterial growth was inhibited by 3-mercaptopropionic acid and aminooxyacetic acid, indicating that these compounds blocked bacterial adaptation to acidic stress. The other three compounds including 4-deoxypyridoxine hydrochloride, isoniazid, and thiosemicarbazide did not inhibit bacterial growth regardless of pH values. To verify the inhibitory effects of 3-mercaptopropionic acid and aminooxyacetic acid, bacterial cells were treated with different concentrations of these compounds from 0 μM to 1,000 μM under acidic pH conditions (Fig. 4B). Bacterial growth was inhibited in proportion to the compound concentrations and aminooxyacetic acid exhibited better inhibitory effect than 3-mercaptopropionic acid. To examine whether these two compounds blocked bacterial adaptation to acid stress by disturbing the GDAR system, their inhibitory effects were re-examined in *E. coli* lacking *gadA* and *gadB*. Addition of 3-mercaptopropionic acid (500 μM) and aminooxyacetic acid (5 μM) decreased the survival ability of wild-type *E. coli* to 23.46 and 16.10%, respectively, during 2 h incubation in pH 2.5 M9 minimal medium broth (Fig. 5). Mutant strains lacking *gadA* or *gadB* suffered from the extreme acid stress, showing strikingly decreased survival levels of less than 20%. However, the decreased survival rates of Δ*gadA* and Δ*gadB* strains under pH 2.5 conditions did not further decline, even with the treatments of 3-mercaptopropionic acid (Fig. 5A) and aminooxyacetic acid (Fig. 5B), indicating that these two compounds exerted the inhibitory effects only in the presence of a GRAD system.

### GDAR System and Copper Resistance Genes as Resistance Factors Against Copper Stress

**Fig. 6:**
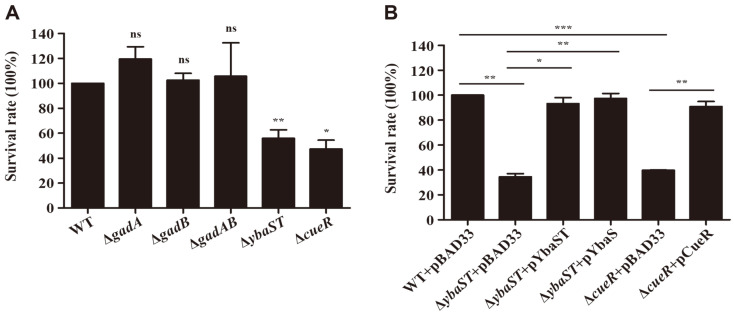
Viability of *E. coli* strains lacking GDAR system and Cue system under copper stress. (**A**) Bacterial strains including wild-type and mutant strains pre-cultured in LB broth overnight were diluted into pH 7.0 M9 minimal medium broth at a 1:100 ratio. The M9 minimal medium broth was supplemented with 50 μM copper or not and the bacterial survival was assayed at 12 h by plating aliquots of serially diluted cultures onto LB agar. Bacterial survival rate was obtained by dividing the number (CFU/ml) from copper-treated sample by the number (CFU/ml) from copper-untreated sample. The survival rate of wild-type *E. coli* was set to 100%. (**B**) Bacterial strains were transformed with pBAD33 or its derivatives expressing *ybaST*, *ybaS*, and *cueR*, respectively, under P_BAD_ promoter. Bacterial strains were incubated in M9 minimal medium broth containing 40 μM copper or not for 12 h as described above and 0.2% arabinose was added to produce YbaST, YbaS, or CueR. Bacterial resistance was compared between 40 μM copper and no treatment and displayed as a relative survival rate where the rate of wild-type *E. coli* was set to 100%. Statistical significance is indicated by *, *p*-value < 0.05; **, *p*-value < 0.01; ***, *p*-value < 0.001.

The transcriptome results revealed that *E. coli* exposed to extreme acid stress increased the transcription of genes encoding the Cue system and the Δ*cueR* mutant strain lacking the regulator of the *cue/cop* regulon was impaired in surviving extreme acid stress, implying interactive cooperation between an acid resistance system and a copper resistance system. To test this possibility, *E. coli* mutant strains with defective GDAR or Cue systems were subjected to copper treatments and vice versa. When the strains were treated with 50 μM copper for 12 h, the survival abilities of *E. coli* strains lacking either *gadA*, *gadB*, or both, were comparable with that of wild-type *E. coli* (Fig. 6A). However, the absence of *ybaST* or *cueR* remarkably decreased bacterial resistance to copper stress and the impaired resistance was completely complemented with the plasmids producing YbaST (pYbaST) or CueR (pCueR) in *trans* (Fig. 6B). Interestingly, the introduction of pYbaS expressing only *ybaS* under P_BAD_ promoter could also recover the attenuated survival of Δ*ybaST* (Fig. 6B). These results demonstrate that YbaST, auxiliary components of the GDAR system, and the Cue system are required for bacterial resistance to both extreme acid and copper stresses.

## Discussion

*E. coli* has acquired multiple resistance systems against acid stresses during its evolutionary adaptation to variable milieux including hostile host defense systems and unfavorable environmental niches. Depending on the strength of the acid stresses, *E. coli* exploits two different resistance mechanisms: acid resistance (AR) systems against extreme acid stress and acid tolerance response (ATR) systems towards moderate acid stress [43]. With regard to AR systems, five (AR1 to AR5) have been identified and four (excluding AR1) harness decarboxylase enzymes to scavenge cytosolic protons using distinct amino acids as substrates, while AR1 system is operated by an alternative sigma factor σ^S^, encoded by *rpoS* [18]. This study revealed that *E. coli* experiencing an extreme acid stress at pH 2.5 promptly increased the expression of AR1 and AR2 systems among the five AR systems (Fig. 2) and the deletion of the genes encoding the AR2 system was detrimental to bacterial resistance against extreme acid stress (Fig. 3), indicating that the GDAR system utilizing glutamate and glutamine is indispensable for *E. coli* survival under extremely acid conditions. The AR2 system, also called the GDAR system, is known to be induced by acid stress but independent of the presence of extracellular glutamate/glutamine, whereas the other three AR systems including AR3, AR4, and AR5 are usually induced by acid stress and the availability of extracellular amino acids [18, 43]. In accordance with previous observations, the attenuated survival of Δ*gadA* and Δ*gadB* under extremely acidic conditions was not altered by the availability of extracellular glutamate (data not shown). The reason that the transcriptome analysis failed to identify the three AR systems (AR3 to AR5) might be attributable to the medium condition used in this study. The transcriptome analysis was conducted using bacterial cells cultured in LB medium broth not supplemented with additional amino acid substrates such as arginine (AR3), lysine (AR4), and ornithine (AR5), thereby not stimulating the expression of these AR systems (Fig. 2A).

The glutamate/glutamine-dependent AR2 system or GDAR system is controlled by a complex hierarchical regulatory cascade comprising multiple regulators that respond to distinct environmental signals [17, 44]. Of these regulators, GadE, GadW, and GadX transcription factors are the first-line regulators and can directly activate or inhibit the transcription of *gadA* and *gadBC*. These master regulators can also activate the transcription of *ybaST* [45, 46] and their binding to the promoter region of *ybaST* was determined using Chip-exo methods [44]. In agreement with the coordinated regulation by GadEWX, genes engaged in the GadEWX regulon, including *gadA*, *gadBC*, *ybaST*, *gadE*, and *gadXW*, showed upregulation in response to pH 2.5 in the transcriptome analysis (Fig. 2). GadE, the central activator for *gadA* and *gadBC*, constitutes the EvgAS-YdeO-GadE regulatory circuit, where EvgA coupled with EvgS senses low pH and triggers the expression of *ydeO* whose product YdeO in turn activates *gadE* during exponential growth in acidic minimal medium [47]. Despite the upregulation of *gadE*, the transcription levels of *evgAS* and *ydeO* were rather decreased in the transcriptome analysis and RT-qPCR assay. This discrepancy might be due to the medium condition tested in this study, where bacterial cells at the stationary phase were treated with a rich medium adjusted to pH 2.5. This result suggests that *gadE* transcription can be stimulated by acidic pH only, independent of EvgAS and YdeO.

YbaS with glutaminase activity is known to contribute to *E. coli* acid resistance by supplementing cytosolic glutamate, the substrate for GadAB, and generating ammonium, thereby neutralizing protons. *ybaT* localized downstream of *ybaS* is supposed to encode an amino acid permease that may supply glutamine to YbaS for hydrolysis [20]. Interestingly, in the *E. coli* genome, the *ybaST* operon is positioned between *copA* and *cueR*, critical determinants for copper resistance, and their transcription is also induced by copper stress [20]. Djoko *et al*. observed that YbaST could confer bacterial resistance to copper stress in the presence of glutamine and speculated about a link between copper stress and acid stress [20]. This study, on the other hand, demonstrated that the *cue/cop* regulon could be stimulated by acid stress and its absence strikingly impaired bacterial survival under extremely acidic conditions (Figs. 2 and 3). To define the complementary roles between acid and copper resistance, mutant strains defective in the GDAR system, including Δ*gadA*, Δ*gadB*, Δ*gadAB*, and Δ*ybaST*, were subjected to copper stress. Of the tested strains, only Δ*ybaST* strain was vulnerable to copper treatments and the attenuated survival was complemented even with YbaS only (Fig. 6). These results reinforced the possibility of cooperative resistance to acid and copper stress via YbaST and the Cue/Cop regulon. Copper is essential for all living organisms but excessive copper can be toxic. Due to its antimicrobial activity, copper is extensively applied to sanitizing coatings on surfaces in hygiene-sensitive areas such as food processing plants and hospitals [48, 49]. *E. coli* employs two different copper resistance systems in response to changes in copper and oxygen availability. The Cue system, which is composed of a copper exporter, CopA, a periplasmic oxidase, CueO, and their cognate copper-sensing regulator CueR, is the primary aerobic apparatus for copper resistance, while, under anaerobic conditions, the supplementary Cus system is required for full copper resistance [33]. The Cus system is a tripartite transporter comprising CusCBA and a two-component regulatory system CusRS activates the expression of cusCFBA operon in response to excessive copper [50]. In contrast to the Cue system, genes encoding the components of the Cus system did not show transcriptional increases under extremely acidic conditions (Fig. 2B), implicating the differential contribution to acid resistance between the Cue and Cus systems. The roles of Cus system in acid resistance remain to be established. Likewise, the mechanism of YbaST-mediated copper resistance should be elucidated.

In conclusion, this study identified a large number of genes that showed altered expression under extremely acidic conditions and could be important for bacterial adaptation to acidic stresses. Of these, the GDAR and Cue systems were found to be indispensable for *E. coli* survival against extreme acid stress. These resistance factors can be exploited as control targets for preventing *E. coli* resistance occurrence to acid treatment, which is commonly used for hygiene purposes in food processing. This approach may also be applicable for controlling other pathogenic bacteria with the GDAR system, such as *Shigella flexneri* and *Listeria monocytogenes*.

## Figures and Tables

**Table 1:** Bacterial strains and plasmids.

Strain/plasmid	Relevant characteristics	Source
*Escherichia coli* strains		
ETEC 4032	Wild-type	Ministry of Food and Drug Safety
MG1655	Wild-type	[21]
Δ*gadA*	MG1655 Δ*gadA*	This study
Δ*gadB*	MG1655 Δ*gadB*	This study
Δ*ybaST*	MG1655 Δ*ybaST*	This study
Δ*cueR*	MG1655 Δ*cueR*	This study
Δ*gadAB*	MG1655 Δ*gadAB*	This study
Plasmids		
pKD46	*bla* P_*BAD*_ *gam beta exo* pSC101 *oriTS*	[22]
pKD13	*bla* FRT *kan* FRT PS1 PS2 *oriR6K*_γ_	[22]
pCP20	*bla cat cI*857 λP_R_ *flp* pSC101 *oriTS*	[22]
pBAD30	pACYC184-ori, Amp^R^, araC, P_*BAD*_	[26]
pBAD18	pBR322-ori, Amp^R^, araC, P_*BAD*_	[26]
pBAD33	pACYC184-ori, Cm^R^, araC, P_*BAD*_	[26]
pGadA	pBAD30::*gadA* Amp^R^	This study
pGadB	pBAD30::*gadB* Amp^R^	This study
pCueR-Amp^R^	pBAD30::*cueR* Amp^R^	This study
pYbaS-Amp^R^	pBAD18::*ybaS* Amp^R^	This study
pYbaST-Amp^R^	pBAD18::*ybaST* Amp^R^	This study
pCueR-Cm^R^	pBAD33::*cueR* Cm^R^	This study
pYbaS-Cm^R^	pBAD33::*ybaS* Cm^R^	This study
pYbaST-Cm^R^	pBAD33::*ybaST* Cm^R^	This study
